# Supplementary material for: Assessing Serum Pepsinogen and Helicobacter pylori Tests for Detecting Diffuse-Type Gastric Cancer: Insights from a Large-Scale and Propensity-Score-Matched Study in Republic of Korea
Source: Cancers (Basel). 2025 Mar 12;17(6):955. doi: 10.3390/cancers17060955 (PMC11940262; doi:10.3390/cancers17060955)
Supplement: Supplementary file 1 [file cancers-17-00955-s001.zip › cancers-3474484-supplementary.pdf]

Supplementary Materials

Supplementary figure.

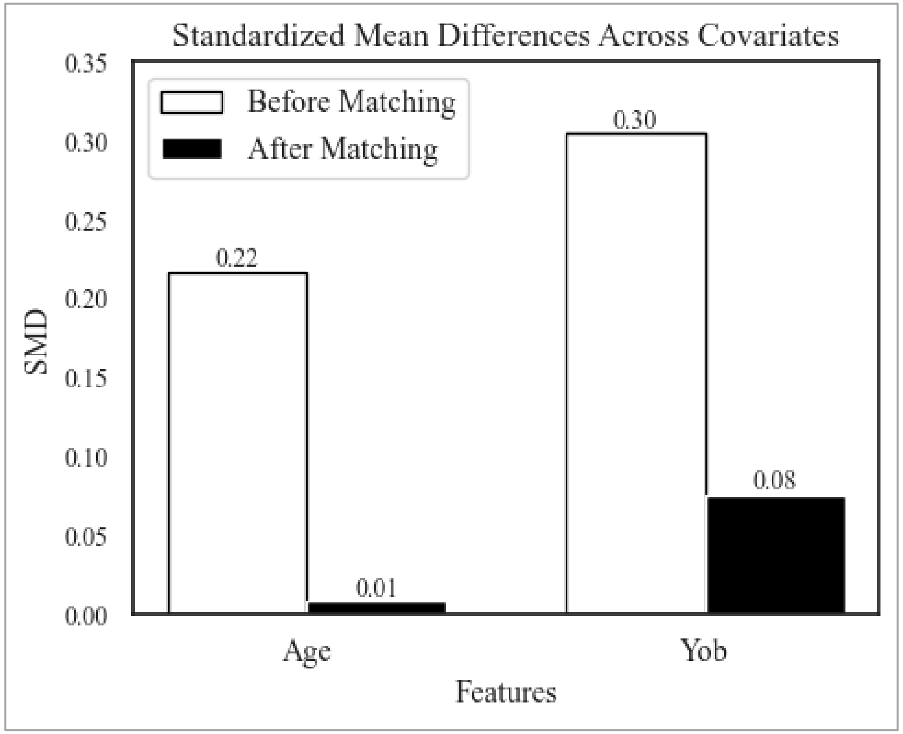

Figure S1. Standardized mean differences plot across covariates

The standardized mean differences (SMD) for age at the time of enrollment (Age) and year of birth (Yob) before and after propensity score matching (PSM). Initially, the covariate Age exhibited an SMD of 0.22, and Yob had an SMD of 0.30, indicating a significant imbalance between the groups. After PSM, the SMDs for both covariates were reduced significantly, demonstrating that PSM effectively mitigated covariate imbalance and thereby enhanced the validity of the causal analysis.

Supplementary tables.

Table S1. Baseline characteristics of the subjects of gastric adenoma before and after propensity-score matching

Characteristics	**Before PSM**			**After PSM**		
	Control	Gastric adenoma	*p* -value	Control	Gastric adenoma	*p* -value
Number (%)	21,320 (92.6)	412 (1.8)		14,724 (92.3)	285 (1.8)	
Sex			0.004			0.478
Female	8,336 (39.1)	132 (32.0)		5,816 (39.5)	97 (34.0)	
Male	12,984 (60.9)	280 (68.0)		8,908 (60.5)	188 (66.0)	
Age, years	54 [13.0]	64 [12.0]	**0.001**	52 [14]	63 [12]	**<0.001**
BMI (kg/m^2)	23.7 [4.0]	23.5 [3.6]	0.678	23.6 [4.0]	23.7 [3.3]	0.559
Smoking			**<0.001**			0.34
Never	10,638 (49.9)	167 (40.5)		7,355 (50.0)	124 (43.5)	
Ever	10,437 (49.0)	244 (59.2)		7,204 (48.9)	160 (56.1)	
Alcohol			0.173			0.936
Never	8,067 (37.8)	143 (34.7)		5,485 (37.3)	101 (35.4)	
Ever	13,163 (61.7)	269 (65.3)		9,170 (62.3)	184 (64.6)	
Salty diet			**<0.001**			**<0.001**
Not/Mild	19,052 (89.4)	295 (71.6)		13,218 (89.8)	210 (73.7)	
Strong	1,909 (8.8)	109 (26.4)		1,243 (8.4)	70 (24.6)	
Family history of GC			**<0.001**			**<0.001**

Negative	18,233 (85.5)	318 (77.2)		12,637 (85.8)	214 (75.1)	
Positive	3,068 (14.4)	94 (22.8)		2,072 (14.1)	71 (24.9)	
HP status			**<0.001**			**<0.001**
Negative	7,811 (36.6)	55 (13.3)		7,715 (52.4)	54 (18.9)	
Positive	13,509 (63.4)	357 (86.7)		7,009 (47.6)	231 (81.1)	
PG series						
PGI, ng/mL	52.3 [27.5]	34.2 [40.8]	**<0.001**	58.2 [25.8]	38.7 [45.3]	**<0.001**
PGII, ng/mL	10.8 [10.1]	12.9 [11.4]	**0.001**	10.0 [8.5]	14.0 [13.0]	**0.002**
PGI/II ratio	4.8 [2.5]	2.6 [2.4]	**<0.001**	5.1 [2.4]	2.7 [2.3]	**<0.001**

n (percentage); median [interquartile range]; GC, gastric cancer; HP, *Helicobacter pylori*; PG, pepsinogen; PGI, serum pepsinogen I; PGII, serum pepsinogen II. Subjects with missing data were shown in Table S1. Values that are statistically significant, with a *p*-value less than 0.05, are displayed in bold. *P*-value* refers to the statistical test between intestinal-type gastric cancer and diffuse-type gastric cancer and was corrected with Bonferroni correction for multiple testing. Salty diet scores: "no/mild" (score 0~2) and "severe" (score 3~4) assessed via two questions ["Do you add extra salt or soy sauce to your food when you eat?" and "Do you eat salty foods such as salted seafood, pickled vegetables, and soup-based meals?" which had three kinds of answers ["never (score 0), sometimes (score 1), frequently (score 2)"], in the questionnaire.

Table S2. Characteristics of gastric cancer by histology

	Intestinal-GC (n=760)	Diffuse-GC (n=490)	*p* - value
**GC stage**			**<0.001**
early	571 (75.1)	239 (48.8)	
advanced	189 (24.9)	251 (51.2)	
**GC location**			0.529
cardia	49 (6.5)	40 (8.2)	
non-cardia	701 (92.2)	440 (89.8)	
both	10 (1.3)	10 (2.0)	
**GC treatment**			**<0.001**
endoscopic	306 (40.3)	31 (6.3)	
surgical	400 (52.7)	380 (77.6)	
chemotherapy	10 (1.3)	27 (5.5)	
conservative	25 (3.3)	36 (7.3)	
unknown[†]	18 (2.4)	16 (3.3)	

Data are presented as number (%). GC, gastric cancer; [†], The patient did not return or was transferred to another hospital and the final treatment was unknown.

Table S3. Subjects with missing data

Variable	Control	Gastric adenoma	GC	Intestinal-GC	Diffuse-GC
**(Before PSM)**	(*n*=21,320)	(*n*=412)	(*n*=1283)	(*n*=760)	(*n*=490)
Smoking	245 (1.1)	1 (0.2)	4 (0.3)	3 (0.4)	1 (0.2)
Alcohol	90 (0.5)	0 (0)	8 (0.6)	5 (0.7)	2 (0.4)
Salty diet	359 (1.7)	8 (2.0)	1 (0.1)	0 (0)	1 (0.1)
Family history of GC	19 (0.1)	0 (0)	1 (0.1)	1 (0.1)	0 (0)
**(After PSM)**	(*n*=14,724)	(*n*=285)	(*n*=945)	(*n*=551)	(*n*=369)
Smoking	165 (1.1)	1 (0.4)	2 (0.2)	2 (0.4)	0 (0)
Alcohol	69 (0.5)	0 (0)	7 (0.7)	5 (0.9)	1 (0.3)
Salty diet	263 (1.8)	5 (1.8)	1 (0.1)	0 (0)	1 (0.3)
Family history of GC	15 (0.1)	0 (0)	1 (0.1)	1 (0.2)	0 (0)

Data are presented as number (%). GC, gastric cancer; PSM, propensity score matching

Table S4. Association between gastric neoplasm and pepsinogens or *Helicobacter pylori* status after propensity-score matching

	GA		GC		IGC		DGC	
	OR (95% CI)	*p* -value	OR (95% CI)	*p* -value	OR (95% CI)	*p* -value	OR (95% CI)	*p* -value
PGI, ng/mL		0.766		**<0.001**		0.861		**<0.001**
>70	1		1		1		1	
≤70	1.04 (0.79-1.38)		0.61 (0.53-0.71)		1.02 (0.83-1.25)		0.35 (0.28-0.43)	
PGII, ng/mL		**<0.001**		**<0.001**		**<0.001**		**<0.001**
<21	1		1		1		1	
≥21	1.96 (1.50-2.56)		3.75 (3.25-4.31)		2.73 (2.26-3.29)		5.51 (4.47-6.79)	
PGR		**<0.001**		**<0.001**		**<0.001**		**<0.001**
>3	1		1		1		1	
≤3	7.24 (5.66-9.25)		5.87 (5.11-6.75)		6.11 (5.09-7.32)		5.72 (4.63-7.08)	
PGIR*		**<0.001**		**<0.001**		**<0.001**		**<0.001**
Negative	1		1		1		1	
Positive	6.77 (5.30-8.64)		4.77 (4.13-5.51)		5.28 (4.05-5.86)		4.33 (3.46-5.42)	
HP status		**<0.001**		**<0.001**		**<0.001**		**<0.001**
Negative	1		1		1		1	
Positive	4.80 (3.55-6.48)		4.04 (3.44-4.73)		3.41 (2.79-4.16)		5.66 (4.42-7.72)	

Adjusted with age and sex; presented as OR (95% CI); PGIR*-positive defined as serum pepsinogen I ≤ 70 ng/mL and pepsinogen ratio ≤ 3, and otherwise, PGIR-negative. GA, gastric adenoma; GC, gastric cancer; IGC, intestinal-type gastric cancer; DGC, diffuse-type gastric cancer; PGI, serum pepsinogen I; PGII, serum pepsinogen II; PGR, PGI/II ratio (pepsinogen ratio); PGIR, serum pepsinogen I & pepsinogen ratio; HP, *Helicobacter pylori*; OR, odds ratio; CI, confidence interval;

Table S5. Multivariable-adjusted logistic regression analysis for subtypes of gastric cancers according to pepsinogen values before and after propensity-score matching

**(Before PSM)**	**PGII, aOR**			**PGIR, aOR**			**PGR, aOR**		
	neg	pos	*p*-value	neg	pos	*p*-value	neg	pos	*p*-value
IGC-E	1	1.46 (1.19-1.79)	**<0.001**	1	3.28 (2.68-4.00)	**<0.001**	1	3.63 (2.98-4.42)	**<0.001**
IGC-A	1	1.68 (1.19-2.38)	**0.003**	1	4.75 (3.37-6.69)	**<0.001**	1	5.48 (3.85-7.81)	**<0.001**
DGC-E	1	5.07 (3.82-6.72)	**<0.001**	1	2.30 (1.73-3.06)	**<0.001**	1	3.83 (2.91-5.05)	**<0.001**
DGC-A	1	1.84 (1.40-2.40)	**<0.001**	1	2.82 (2.14-3.71)	**<0.001**	1	3.35 (2.56-4.39)	**<0.001**
**(After PSM)**	**PGII, aOR**			**PGIR, aOR**			**PGR, aOR**		
	neg	pos	*p*-value	neg	pos	*p*-value	neg	pos	*p*-value
IGC-E	1	1.01 (0.75-1.36)	0.954	1	1.28 (0.76-1.82)	0.285	1	2.89 (1.79-4.65)	**<0.001**
IGC-A	1	1.35 (0.85-2.14)	0.202	1	1.59 (0.81-3.15)	0.179	1	3.13 (1.51-6.49)	**0.002**
DGC-E	1	4.20 (2.87-6.14)	**<0.001**	1	1.04 (0.65-1.65)	0.884	1	1.87 (1.16-3.03)	**0.01**
DGC-A	1	1.37 (0.96-1.95)	0.084	1	1.71 (0.99-2.96)	0.054	1	1.93 (1.08-3.43)	**0.026**

Multivariables adjusted for sex, age, body mass index, family history of gastric cancer, salty food, smoking, alcohol intake, and *Helicobacter pylori* status. Values that are statistically significant, with a *P*-value of < 0.05, are displayed in bold. PGII neg (PGII- negative), serum pepsinogen II < 21ng/ml; PGII pos (PGII- positive), serum pepsinogen II ≥ 21ng/ml; PGIR neg (PGIR-negative), serum pepsinogen I > 70 or pepsinogen ratio > 3; PGIR pos (PGIR-positive), serum pepsinogen I ≤ 70 and pepsinogen ratio ≤ 3; PGR neg (PGR-negative), pepsinogen ratio > 3; PGR pos (PGR-positive), pepsinogen ratio ≤ 3; aOR, adjusted odds ratio; IGC-E, intestinal-type gastric cancer, early-stage; IGC-A, intestinal-type gastric cancer, advanced stage; DGC-E, diffuse-type gastric cancer, early stage; DGC-A, diffuse-type gastric cancer, advanced stage

Table S6. Positive predictive value of each pepsinogen value for the diagnosis of gastric cancer

		PGII-positive	PGR-positive	PGII+HP-positive	PGR+HP-positive
Gastric neoplasm	PPV (95% CI)	13.3 (12.3-14.3)	19.0 (18.0-20.2)	13.1 (12.2-14.2)	18.8 (17.7-20.0)
	NPV (95% CI)	94.6 (94.2-94.9)	96.2 (95.9-96.5)	94.4 (94.1-94.8)	95.9 (95.6-96.1)
Gastric cancer	PPV (95% CI)	11.4 (10.5-12.3)	15.1 (14.1-16.2)	11.2 (10.3-12.2)	14.9 (13.8-16.0)
	NPV (95% CI)	96.0 (95.7-96.3)	97.0 (96.7-97.2)	95.9 (95.6-96.1)	96.7 (96.4-96.9)
Intestinal-gastric cancer	PPV (95% CI)	5.8 (5.1-6.5)	9.4 (8.6-10.3)	5.7 (5.0-6.5)	9.2 (8.3-10.1)
	NPV (95% CI)	97.3 (97.1-97.6)	98.3 (98.1-98.4)	97.3 (97.0-97.5)	98.1 (97.9-98.3)
Diffuse- gastric cancer	PPV (95% CI)	5.5 (4.8-6.2)	5.7 (5.0-6.4)	5.4 (4.8-6.2)	5.7 (5.0-6.4)
	NPV (95% CI)	98.6 (98.5-98.8)	98.7 (98.5-98.9)	98.6 (98.4-98.7)	98.6 (98.5-98.8)

PG, pepsinogen; HP, *Helicobacter pylori* status; PGII-positive, serum pepsinogen II ≥ 21 ng/mL; PGR-positive, pepsinogen ratio ≤ 3; PGII+HP-positive, serum pepsinogen II ≥ 21 ng/mL and *Helicobacter pylori* status positive; PGR+HP-positive, pepsinogen ratio ≤ 3 and *Helicobacter pylori* status positive; Gastric neoplasm, gastric adenoma and gastric cancer; PPV, positive predictive value; NPV, negative predictive value; CI, confidence interval

Table S7. Risk stratification by combining pepsinogen II and *Helicobacter pylori* status in diffuse-type gastric cancer after propensity-score matching

Variable	HP / PGII	DGC-T			DGC-E			DGC-A		
		n/N	OR (95%CI)	*p*-value	n/N	OR (95%CI)	*p*-value	n/N	OR (95%CI)	*p*-value
Total	(-) / (-)	45/7,620	1		20/7,645	1		25/7,640	1	
	(+) / (-)	142/5,285	1.18 (0.96-1.46)	0.122	49/5,378	0.72 (0.52-1.01)	0.056	93/5,334	1.76 (1.33-2.34)	**<0.001**
	(-) / (+)	15/231	2.76 (1.62-4.70)	**<0.001**	8/238	3.05 (1.48-6.26)	**0.002**	7/239	2.40 (1.11-5.15)	**0.025**
	(+) / (+)	167/2,139	5.08 (4.12-6.26)	**<0.001**	99/2,207	7.73 (5.72-10.44)	**<0.001**	68/2,238	3.21 (2.38-4.33)	**<0.001**
Male ≥40 yr	(-) / (-)	23/3,910	1		9/3,924	1		14/3,919	1	
	(+) / (-)	79/3,134	1.30 (0.96-1.74)	0.088	24/3,189	0.74 (0.46-1.21)	0.231	55/3,158	1.90 (1.29-2.81)	**0.001**
	(-) / (+)	9/151	2.85 (1.43-5.68)	**0.003**	4/156	2.91 (1.05-8.05)	**0.04**	5/155	2.71 (1.09-6.76)	**0.032**
	(+) / (+)	69/1,129	3.96 (2.91-5.38)	**<0.001**	40/1,158	6.79 (4.32-10.66)	**<0.001**	29/1,169	2.42 (1.57-3.74)	**<0.001**
Female ≥40 yr	(-) / (-)	17/2,969	1		9/2,977	1		8/2,978	1	
	(+) / (-)	48/1,564	1.32 (0.92-1.88)	0.128	20/1,592	0.87 (0.52-1.46)	0.61	28/1,584	2.04 (1.23-3.37)	**0.006**
	(-) / (+)	6/71	3.43 (1.46-8.03)	**0.005**	4/73	4.12 (1.47-11.58)	**0.007**	2/75	2.41 (0.58-10.06)	0.226
	(+) / (+)	67/825	5.27 (3.74-7.41)	**<0.001**	43/849	7.12 (4.50-11.28)	**<0.001**	24/868	3.37 (2.01-5.65)	**<0.001**
Male <40 yr	(-) / (-)	2/470	1		1/471	1		1/471	1	
	(+) / (-)	7/401	1.05 (0.40-2.79)	0.918	3/405	0.90 (0.21-3.79)	0.887	4/404	1.20 (0.32-4.51)	0.783
	(-) / (+)	0/9	nc		0/9	nc		0/9	nc	
	(+) / (+)	8/124	6.31 (2.39-16.65)	**<0.001**	4/128	6.91 (1.71-27.99)	**0.007**	4/128	5.52 (1.46-20.84)	**0.012**
Female <40 yr	(-) / (-)	3/271	1		1/273	1		2/272	1	
	(+) / (-)	8/186	0.55 (0.24-1.24)	0.148	2/192	0.28 (0.06-1.24)	0.093	6/188	0.85 (0.32-2.26)	0.741
	(-) / (+)	0/0	nc		0/0	nc		0/0	nc	
	(+) / (+)	23/61	15.66 (7.28-33.72)	**<0.001**	12/72	25.83 (7.12-93.78)	**<0.001**	11/73	8.66 (3.37-22.26)	**<0.001**

HP, *Helicobacter pylori* status; PGII, serum pepsinogen II; DGC-T, diffuse-type gastric cancer, total; n, number of cases; N, number of controls; OR, odds ratio; CI, confidence intervals; DGC-E, diffuse-type early-gastric cancer; DGC-A, diffuse-type advanced-gastric cancer; HP (-), HP status negative; HP (+), HP status positive; PGII (+), serum pepsinogen II ≥ 21 ng/mL; PGII (-), serum pepsinogen II 〈 21 ng/mL; yr, years old; nc, noncomputable;

Table S8. Risk stratification by combining pepsinogen ratio and *Helicobacter pylori* status in diffuse-type gastric cancer after propensity score matching

Variable	HP / PGR	DGC-T			DGC-E			DGC-A		
		n/N	OR (95%CI)	*p*-value	n/N	OR (95%CI)	*p*-value	n/N	OR (95%CI)	*p*-value
Total	(-) / (-)	40/7,545	1		21/7,564	1		19/7,566	1	
	(+) / (-)	148/5,344	1.24 (1.01-1.54)	**0.042**	68/5,424	1.17 (0.86-1.58)	0.324	80/5,412	1.31 (0.98-1.75)	0.064
	(-) / (+)	20/306	2.80 (1.76-4.46)	**<0.001**	7/319	1.97 (0.92-4.22)	0.083	13/313	3.49 (1.97-6.20)	**<0.001**
	(+) / (+)	161/2,080	4.91 (3.98-6.06)	**<0.001**	80/2,161	5.13 (3.80-6.93)	**<0.001**	81/2,160	4.45 (3.33-5.94)	**<0.001**
Male ≥40 yr	(-) / (-)	17/3,900	1		9/3,908	1		8/3,909	1	
	(+) / (-)	70/3,195	1.02 (0.75-1.38)	0.89	29/3,236	0.97 (0.61-1.54)	0.895	41/3,224	1.06 (0.71-1.58)	0.767
	(-) / (+)	15/161	4.61 (2.66-8.00)	**<0.001**	4/172	2.63 (0.95-7.28)	0.063	11/165	5.97 (3.13-11.36)	**<0.001**
	(+) / (+)	78/1,068	5.20 (3.84-7.03)	**<0.001**	35/1,111	5.49 (3.49-8.63)	**<0.001**	43/1,103	4.74 (3.19-7.05)	**<0.001**
Female ≥40 yr	(-) / (-)	18/2,907	1		10/2,915	1		8/2,917	1	
	(+) / (-)	48/1,510	1.38 (0.97-1.97)	0.073	27/1,531	1.43 (0.89-2.29)	0.142	21/1,537	1.32 (0.78-2.24)	0.301
	(-) / (+)	5/133	1.50 (0.60-3.72)	0.384	3/135	1.63 (0.51-5.24)	0.412	2/136	1.32 (0.32-5.44)	0.705
	(+) / (+)	67/879	4.88 (3.47-6.87)	**<0.001**	36/910	4.53 (2.87-7.15)	<0.001	31/915	5.02 (3.03-8.29)	**<0.001**
Male <40 yr	(-) / (-)	2/468	1		1/469	1		1/469	1	
	(+) / (-)	13/449	4.02 (1.30-12.41)	**0.016**	6/456	3.66 (0.74-18.24)	0.113	7/455	4.28 (0.89-20.73)	0.07
	(-) / (+)	0/11	nc		0/11	nc		0/11	nc	
	(+) / (+)	2/76	1.63 (0.37-7.25)	0.523	1/77	1.74 (0.21-14.30)	0.608	1/77	1.52 (0.19-12.29)	0.696
Female <40 yr	(-) / (-)	3/270	1		1/272	1		2/271	1	
	(+) / (-)	17/190	1.73 (0.86-3.46)	0.124	6/201	1.11 (0.39-3.18)	0.839	11/196	2.36 (0.94-5.98)	0.069
	(-) / (+)	0/1	nc		0/1	nc		0/1	nc	
	(+) / (+)	14/57	5.66 (2.71-11.82)	**<0.001**	8/63	8.60 (3.02-24.52)	**<0.001**	6/65	3.32 (1.22-9.05)	**0.019**

HP, *Helicobacter pylori* status; PGR, pepsinogen ratio; DGC-T, diffuse-type gastric cancer, total; n, number of cases; N, number of controls; OR, odds ratio; CI, confidence intervals; DGC-E, diffuse-type early-gastric cancer; DGC-A, diffuse-type advanced-gastric cancer; HP (-), HP status negative; HP (+), HP status positive;PGR (+), pepsinogen ratio ≤ 3; PGR (-), pepsinogen ratio 〉 3; yr, years old; nc, noncomputable

Table S9. Risk stratification by combining pepsinogen II and *Helicobacter pylori* status in intestinal-type gastric cancer after propensity-score matching

Variable	HP / PGII	IGC-T			IGC-E			IGC-A		
		n/N	OR (95%CI)	*p*-value	n/N	OR (95%CI)	*p*-value	n/N	OR (95%CI)	*p*-value
Total	(-) / (-)	118/7,547	1		86/7,579	1		32/7,633	1	
	(+) / (-)	245/5,182	1.53 (1.29-1.82)	**<0.001**	190/5,237	1.70 (1.40-2.08)	**<0.001**	55/5,372	1.11 (0.80-1.56)	0.526
	(-) / (+)	18/228	2.20 (1.35-3.59)	**0.002**	15/231	2.51 (1.48-4.28)	**0.001**	3/243	1.30 (0.41-4.10)	0.656
	(+) / (+)	170/2,136	2.71 (2.25-3.26)	**<0.001**	112/2,194	2.29 (1.83-2.86)	**<0.001**	58/2,248	3.80 (2.72-5.30)	**<0.001**
Male	(-) / (-)	85/2,472	1		60/2,497	1		25/2,532	1	
≥50 yr	(+) / (-)	169/1,883	1.43 (1.16-1.77)	**0.001**	130/1,922	1.74 (1.35-2.23)	**<0.001**	39/2,013	0.88 (0.60-1.31)	0.535
	(-) / (+)	13/117	1.55 (0.87-2.78)	0.139	10/120	1.72 (0.89-3.31)	0.108	3/127	1.13 (0.35-3.60)	0.837
	(+) / (+)	108/701	2.58 (2.04-3.27)	**<0.001**	61/748	1.85 (1.38-2.49)	**<0.001**	47/762	4.30 (2.94-6.29)	**<0.001**
Female	(-) / (-)	27/2,098	1		20/2,105	1		7/2,118	1	
≥50 yr	(+) / (-)	46/1,072	1.77 (1.21-2.59)	**0.003**	35/1,083	1.73 (1.12-2.67)	**0.014**	11/1,107	1.87 (0.85-4.08)	0.117
	(-) / (+)	5/61	2.87 (1.13-7.27)	**0.027**	5/61	3.80 (1.49-9.71)	**0.005**	0/66	na	
	(+) / (+)	35/606	2.39 (1.59-3.60)	**<0.001**	27/614	2.38 (1.50-3.78)	**<0.001**	8/633	2.31 (1.00-5.34)	**0.05**
Male	(-) / (-)	3/1,845	1		3/1,845	1		0/1,848	NA	
<50 yr	(+) / (-)	23/1,546	1.61 (0.90-2.90)	0.111	18/1,551	1.46 (0.76-2.79)	0.253	5/1,564		
	(-) / (+)	0/39	nc		0/39	nc		0/39		
	(+) / (+)	19/502	4.99 (2.74-9.09)	**<0.001**	16/505	5.18 (2.69-10.00)	**<0.001**	3/518		
Female	(-) / (-)	3/1,132	1		3/1,132	1		0/1,135	NA	
<50 yr	(+) / (-)	7/681	1.37 (0.53-3.56)	0.513	7/681	1.37 (0.53-3.56)	0.513	0/688		
	(-) / (+)	0/11	nc		0/11	nc		0/11		
	(+) / (+)	8/327	4.46 (1.75-11.39)	**0.002**	8/327	4.46 (1.75-11.39)	**0.002**	0/335		

HP, *Helicobacter pylori* status; PGII, serum pepsinogen II; IGC-T, intestinal-type gastric cancer, total; n, number of cases; N, number of controls; OR, odds ratio; CI, confidence intervals; IGC-E, intestinal-type early-gastric cancer; IGC-A, intestinal-type advanced-gastric cancer; HP (-), HP status negative; HP (+), HP status positive; PGII (+), serum pepsinogen II ≥ 21 ng/mL; PGII (-), serum pepsinogen II 〈 21 ng/mL; yr, years old; nc, noncomputable; NA, not applicable

Table S10. Risk stratification by combining pepsinogen ratio and *Helicobacter pylori* status in intestinal-type gastric cancer after propensity-score matching

**Variable**	**HP / PGR**	**IGC-T**			**IGC-E**			**IGC-A**		
		n/N	OR (95%CI)	*p*-value	n/N	OR (95%CI)	*p*-value	n/N	OR (95%CI)	*p*-value
Total	(-) / (-)	97/7,488	1		71/7,514	1		26/7,559	1	
	(+) / (-)	154/5,338	0.71 (0.59-0.86)	**<0.001**	122/5,370	0.80 (0.64-0.99)	**0.04**	32/5,460	0.51 (0.34-0.75)	**0.001**
	(-) / (+)	39/287	3.93 (2.78-5.55)	**<0.001**	30/296	4.06 (2.75-5.99)	**<0.001**	9/317	3.10 (1.57-6.14)	**0.001**
	(+) / (+)	261/1,980	5.96 (5.01-7.09)	**<0.001**	180/2,061	5.16 (4.22-6.32)	**<0.001**	81/2,160	7.46 (5.38-10.35)	**<0.001**
Male	(-) / (-)	67/2,467	1		46/2,488	1		21/2,513	1	
≥50 yr	(+) / (-)	100/1,889	0.63 (0.50-0.80)	**<0.001**	75/1,914	0.71 (0.54-0.93)	**0.014**	25/1,964	0.50 (0.32-0.78)	**0.002**
	(-) / (+)	31/122	3.73 (2.48-5.62)	**<0.001**	24/129	4.05 (2.57-6.38)	**<0.001**	7/146	2.37 (1.08-5.18)	**0.031**
	(+) / (+)	177/695	5.76 (4.63-7.16)	**<0.001**	116/756	4.79 (3.71-6.19)	**<0.001**	61/811	6.56 (4.51-9.55)	**<0.001**
Female	(-) / (-)	24/2,061	1		19/2,066	1		5/2,080	1	
≥50 yr	(+) / (-)	27/1,034	0.85 (0.55-1.32)	0.471	23/1,038	0.98 (0.60-1.58)	0.928	4/1,057	0.49 (0.17-1.43)	0.194
	(-) / (+)	8/98	2.91 (1.38-6.13)	**0.005**	6/100	2.79 (1.19-6.54)	**0.018**	2/104	3.06 (0.71-13.12)	0.132
	(+) / (+)	54/644	4.54 (3.11-6.63)	**<0.001**	39/659	3.95 (2.57-6.08)	**<0.001**	15/683	6.47 (2.96-14.15)	**<0.001**
Male	(-) / (-)	3/1,850	1		3/1,850	1		0/1,853	NA	
<50 yr	(+) / (-)	20/1,718	1.03 (0.57-1.86)	0.919	17/1,721	1.10 (0.57-2.10)	0.782	3/1,735		
	(-) / (+)	0/34	nc		0/34	nc		0/34		
	(+) / (+)	22/330	10.44 (5.76-18.93)	**<0.001**	17/335	9.15 (4.75-17.63)	**<0.001**	5/347		
Female	(-) / (-)	3/1,110	1		3/1,110	1		0/1,113	NA	
<50 yr	(+) / (-)	7/697	1.33 (0.51-3.44)	0.56	7/697	1.33 (0.51-3.44)	0.56	0/704		
	(-) / (+)	0/33	nc		0/33	nc		0/33		
	(+) / (+)	8/311	4.73 (1.85-12.09)	**0.001**	8/311	4.73 (1.85-12.09)	**0.001**	0/319		

HP, *Helicobacter pylori* status; PGR, pepsinogen ratio; IGC-T, intestinal-type gastric cancer, total; n, number of cases; N, number of controls; OR, odds ratio; CI, confidence intervals; IGC-E, intestinal-type early-gastric cancer; IGC-A, intestinal-type advanced-gastric cancer; HP (-), HP status negative; HP (+), HP status positive; PGR (+), pepsinogen ratio ≤ 3; PGR (-), pepsinogen ratio 〉 3; yr, years old; nc, noncomputable; NA, not applicable

Table S11. Simulation of diagnostic power depending on age and sex

	Real data		Simulation											
	**total number**	**GC number**	No. of performing EGD	No. of GC-diagnosed	No. of not performing EGD	No. of GC-undiag-nosed	Diagnostic power: Sensiti-vity	Specifi-city	PPV	NPV	FPR	FNR	Accur-acy	Detection rate of GC†
< 40Y	**1690**	**63**	857	57	833	6	90.48	50.83	6.65	99.28	49.17	9.52	52.31	3.37
< 40Y F	**601**	**41**	284	37	317	4	90.24	55.89	13.03	98.74	44.11	9.76	58.24	6.16
< 40Y M	**1089**	**22**	573	20	516	2	90.91	48.17	3.49	99.61	51.83	9.09	49.04	1.84
≥ 40Y	**21325**	**1220**	14601	1094	6724	126	89.67	32.82	7.49	98.13	67.18	10.33	36.07	5.13

< 40Y, under 40 years old; < 40Y F, female under 40 years old; < 40Y M, male under 40 years old; ≥ 40Y, 40 years old and older, GC, gastric cancer, No., number; EGD, esophagogastroduodenoscopy; PPV, positive predictive value; NPV, negative predictive value; FPR, false positive rate; FNR, false negative rate; †, percentage of (No. of GC diagnosed / total number), this calculation formula is obtained from reference 49.
